# Genetics and Therapeutics in Pediatric Ulcerative Colitis: the Past, Present and Future

**DOI:** 10.12688/f1000research.7440.1

**Published:** 2016-02-29

**Authors:** Luis Sifuentes-Dominguez, Ashish S. Patel

**Affiliations:** 1 Children's Health, UT Southwestern Medical Center, Dallas, Texas, USA

**Keywords:** Ulcerative colitis, genetic risk factors, therapeutics, personalized treatment strategies

## Abstract

Ulcerative colitis (UC) is a relapsing and remitting disease with significant phenotypic and genotypic variability. Though more common in adults, UC is being increasingly diagnosed in childhood. The subsequent lifelong course of disease results in challenges for the patient and physician. Currently, there is no medical cure for UC. Even though surgical removal of the colon can be curative, complications including infertility in females make colectomy an option often considered only when the disease presents with life-threatening complications or when medical management fails. One of the greatest challenges the clinician faces in the care of patients with UC is the inability to predict at diagnosis which patient is going to respond to a specific therapy or will eventually require surgery. This therapeutic conundrum frames the discussion to follow, specifically the concept of individualized or personalized treatment strategies based on genetic risk factors. As we move to therapeutics, we will elucidate traditional approaches and discuss known and novel agents. As we look to the future, we can expect increasing integrated approaches using several scientific disciplines to inform how genetic interactions shape and mold the pathogenesis and therapeutics of UC.

Ulcerative colitis (UC) is a chronic relapsing and remitting disease characterized by inflammation, ulceration, and bleeding in the large intestine. UC can present with significant phenotypic and genetic variability. This variability results in substantial difficulty as it relates to the appropriate therapeutic approach for an individual patient. We will first investigate the concept of individualized or personalized treatment strategies by describing the current understanding of genetics in pediatric UC. We will then move to the therapeutic discussion, focusing on traditional approaches, known agents in new roles, and novel agents and targets. Our intent will be to frame the therapeutic discoveries of the past, present, and future on the timeline of genetic discovery.

## Genetics of pediatric ulcerative colitis

Evolving technologies are allowing us to dissect the genetic architecture of inflammatory bowel disease (IBD) ( Figure 1). Early epidemiological observations served as the basis for a genetic model of IBD, and a growing body of evidence continues to support and strengthen this notion. Genetic discoveries over the past century have greatly advanced our understanding of UC pathogenesis. These discoveries have provided great insight into disease-associated pathways and have had a dramatic influence over drug discovery and development.

Interestingly, Crohn’s disease (CD) and UC share a large number of the described susceptibility loci, suggesting that they may share a common genetic background. Clinical differences, however, would suggest that different genes and pathways are involved in each disease and thus these differences may explain the discrepant phenotypes and may certainly provide the basis for targeted drug development.

## Genetic epidemiology

Clinical recognition of disease clustering in select families has long been appreciated ^1, 2^. Estimated IBD prevalence among family members of patients with UC is 7.9% to 12% ^3, 4^, with high concordance for disease type. Although these observations may be explained by shared environmental factors in family members, further proof of genetic involvement has come from twin studies that show higher concordance rates of IBD among monozygotic twins. A recent analysis of available twin studies showed a 15.4% concordance rate for UC among monozygotic twins compared to 3.9% in dizygotic counterparts ^5^. Yet UC does not exhibit traditional Mendelian inheritance and thus is a complex polygenic disease.

IBD clusters not only in families but also among populations. IBD prevalence is significantly higher in whites than in non-whites, notably in those of European ancestry ^6^. Ethnic aggregation is particularly seen in Ashkenazi Jews in whom disease prevalence is highest ^7, 8^. On the other hand, disease incidence continues to increase worldwide ^9^, particularly in westernized societies, enforcing the concept that gene-environment interactions are at the core of IBD pathogenesis.

## Linkage studies

In the latter part of the 20 ^th^ century, association and linkage disequilibrium studies yielded fruitful evidence of loci associated with IBD susceptibility. The first IBD susceptibility locus was described in 1996 ^10^ and mapped to chromosome 16, where *NOD2* would later be identified as a candidate CD-associated gene ^11, 12^. Similar studies followed and genetic heterogeneity of both CD and UC was quickly appreciated. It became apparent that many genes may also participate in the phenotypic expression of UC.

To overcome the limitations of narrow genome coverage of linkage disequilibrium studies in the analysis of polygenic diseases, several key developments would have to occur. As pointed out by Mathew ^13^, the creation of dense single nucleotide polymorphism (SNP) libraries, the development of a high-resolution genetic map that allowed measurement of association between SNPs (HapMap), and the advent of high-throughput genotyping methods provided the necessary tools for further discovery of IBD susceptibility loci through genome-wide association studies (GWAS).

## The Genome-Wide Association Study era

To study polygenic diseases, GWAS rely on the premise that genome-wide genetic variations that may be associated with a specific disease will occur with greater frequency in subjects with the disease when compared to healthy controls. These associations must withstand rigorous statistical methods. Because signals from individual loci may be very small, GWAS require large patient and control cohorts to establish significance.

More than any other complex polygenic disease, IBD has been the subject of numerous GWAS and meta-analyses ^14– 24^. Results from these studies have helped unravel the inner workings of IBD pathophysiology and numerous genes affecting key pathways have been described (relevant pathways discussed below). More recently, meta-analyses combining GWAS and Immunochip data (genotyping chip containing >196,000 polymorphisms) ^25^ have dramatically increased the number of IBD-associated loci.

The first publication using this strategy combined data from 75,000 samples ^26^. This study expanded the known IBD susceptibility loci to 163, with 23 of them UC specific and 110 (67%) shared by both CD and UC. Three important observations were also made by this study. First, IBD shows overlap with other immune-related diseases such as primary immunodeficiencies, spondyloarthropathies, and psoriasis, suggesting a common origin and pathophysiological basis. Second, IBD loci were significantly enriched in genes implicated in susceptibility to mycobacterial diseases, perhaps conserved by evolutionary pressure. Third, a large proportion of loci were found in non-coding regions, adding fuel to the idea that non-coding variation may be responsible for differential expression of complex traits.

A follow-up study using a similar approach incorporated more than 96,000 samples from European, East Asian, Indian, and Iranian populations, increasing the number of susceptibility loci to 200 ^27^. Of notable interest was the finding that most loci were shared by all populations studied, implying that causal variants are common, even in ethnically diverse individuals. Admixed populations, such as African-Americans, have also recently been scrutinized using the Immunochip, and previously established loci were replicated in this population as well ^28^.

## Current state of ulcerative colitis genetics

A common finding among GWAS and meta-analyses is increased signal enrichment in regions that participate in intestinal immune homeostasis. Functional characterization of genes found in these loci has led to important discoveries in the pathways involved in disease. Below, we describe relevant genes implicated in pathways associated with UC.

### HLA genes

Because of their roles in antigen presentation and T-cell priming, HLA gene variants have long been considered prime candidates for IBD susceptibility ^29, 30^. Indeed, pre-GWAS era linkage disequilibrium studies first described genetic association of IBD susceptibility to major histocompatibility complex (MHC) regions. Hampered by difficulties inherent to the linkage disequilibrium architecture of the area, susceptibility loci identification was slow ^31^. However, all GWAS to date have continued to report a strong association between HLA loci and UC. A recent study performed high-density mapping of the MHC region in over 32,000 patients with IBD ^32^. This study found significant enrichment in MHC class II alleles: HLA DRB1, HLA DQA1, and HLA-DRB1*01:03 in particular were strongly associated with UC. The HLA-DRB1*01:03 allele is of particular interest as it also showed a strong association with CD colitis and other studies have linked it to pancolitic phenotype in UC ^33^. Goyette also found decreased class II HLA heterozygosity, suggesting that broader colonic antigen recognition is important in protection against UC development.

These associations are especially intriguing for pediatric IBD, since young patients, in particular those less than 8 years of age, are more prone to developing extensive colonic disease ^34^. Two pediatric IBD GWAS studies have replicated findings of HLA polymorphisms with UC susceptibility ^17, 18^.

### Barrier function

Immune tolerance to food and the normal intestinal microbiota is crucial for the control of intestinal homeostasis. A critical element in maintaining intestinal immune homeostasis is epithelial integrity. In addition, the observation that *MUC2 ^-/-^* deficient mice develop spontaneous colitis ^35^ suggests that the intestinal mucus layer contributes to barrier function and colonic immune homeostasis. Epithelial barrier function in UC is impaired ^36^ and active UC is characterized by mucin depletion.

Not surprisingly, polymorphisms associated with UC have been found in genes controlling key aspects of barrier function. These include polymorphisms in *ECM1*, believed to control epithelial basement membrane integrity ^37, 38^. SNPs in the CDH1 locus, encoding the adherens junction protein E-cadherin required for tight junction formation, *HNF4a*, responsible for epithelial differentiation, and *LAMB1*, encoding the β1 subunit of basement membrane laminin, have also been identified ^14^ and replicated ^39^. Finally, polymorphisms in *GNA12*, associated with tight junction assembly via interactions with Zo-1 and Src, have recently been described ^40^.

### Unfolded protein response

The unfolded protein response (UPR) is a stress response of the endoplasmic reticulum (ER) aimed at maintaining cellular homeostasis, especially in highly secretory cells such as Paneth cells and goblet cells. ER stress has been linked to environmental influences and loss or dysregulation of the UPR leads to activation of apoptotic pathways. The intestinal surface is the largest epithelium in contact with the environment, so it is not surprising that genes controlling the UPR pathway have been associated with IBD pathogenesis. Bertolotti *et al.* reported that mice lacking IRE1β expression, a component of the UPR, develop exacerbated dextran sulfate sodium (DSS)-induced colitis ^41^. Human UPR-associated polymorphisms have also been described. Kaser *et al.* reported associations of *XBP1* variants, a transcription factor involved in the UPR, with development of UC ^42^. Similarly, susceptibility-associated variants in *PTPN2*, a protein tyrosine phosphatase linked to UPR and autophagosome formation ^43, 44^, have also been described ^40, 45^.

### Interleukin-23

The interleukin (IL)-23 pathway plays a key role in expansion and differentiation of T helper (T _h_) and innate immune cells. Abnormalities in the IL-23 signaling pathway have been linked to development of UC in several cohorts ^23^. Other components of the pathway such as JAK2 and TYK2 have also been linked to disease pathogenesis ^37^. The importance of this pathway in regulating pro-inflammatory effects seen in IBD is highlighted by the clinical improvement seen in patients with CD when treated with ustekinumab, a monoclonal antibody directed against the p40 subunit shared by IL-12 and IL-23 ^46^. The ongoing phase 3 international multicenter study UNIFI aims to evaluate this monoclonal antibody in maintenance and induction of patients with moderate to severe UC ^47^.

Other T _h_17-centric polymorphisms have been associated with UC, including those in *NFKBIZ,* a regulator of T _h_17 cell development, and *AHR*, a transcription factor involved in T _h_17 cytokine expansion ^27^. Additional important polymorphisms in blocks containing immune regulatory genes have been described. For a detailed review please refer to the article by Khor *et al.* ^48^.

## Genome-Wide Association Study limitations and missing heritability

Results from meta-analyses employing Immunochip indicate that the expected UC disease variance from susceptibility loci is only 8.2%. Therefore, there is a wide gap in the expected disease heritability, a problem termed “missing heritability”. One approach to tackling the missing heritability problem in IBD is the study of patients with single gene disorders with large effects. These monogenic forms of IBD are highly penetrant and cluster in young children, the very early onset IBD group (VEOIBD). Indeed, numerous single gene disorders have been recently described and include mutations in *ADAM17, NCF2, NCF4, TTC7A, IL-10,* and *XIAP* ^49– 54^.

### Next-generation sequencing

Although GWAS have dramatically influenced our understanding of complex diseases, one limitation of such studies is that they neglect contribution from rare variants, those with minor allele frequencies less than 5%. High effect rare risk variants missed by GWAS may be contained within known IBD susceptibility loci. Two studies have sought to identify causal functionally relevant genetic variants contained within IBD susceptibility loci by employing deep next-generation sequencing. Results from these studies pointed to three new mutations associated with UC. While variants in *CARD9* and *IL23R* were protective, a variant in *RNF186*, a RING ubiquitin ligase, was found to confer risk ^55, 56^. In addition to reporting novel associations with IBD risk, these studies provide new insight into future methodological strategies for gene discovery in IBD.

**Figure 1.**
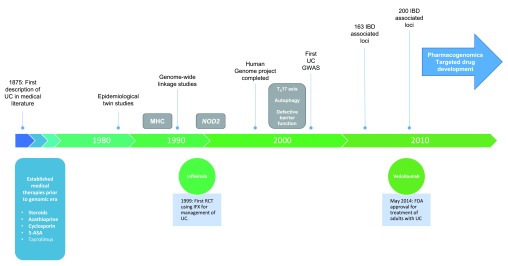
Timeline of genetic discoveries and recent drug developments in IBD. *Adapted from* Expert Rev Gastroenterol Hepatol. 2009 513–34.

## Implications for translation

One of the most promising aspects of genetics is the use of genetic markers to help inform clinical decision-making. In this regard, IBD genetics has made significant strides.

### Diagnosis

Currently, pediatric UC diagnosis is based on aggregate clinical, endoscopic, imaging, and histopathological findings as suggested by North American and European working groups ^57, 58^. The rapid pace of discovery of UC-associated genetic variants suggests that genetic diagnosis is quickly unfolding. To date, UC-specific diagnostic approaches utilizing genetic expression analysis from colonic biopsies ^59, 60^, composite genetic risk scores ^61^, and combined serological and genetic markers ^62^ have shown promise, yet application of these tools has yet to invade day-to-day clinical practice. The VEOIBD age group is an exception. As monogenic forms of IBD cluster in this age group, exome and targeted gene chips are increasingly being suggested and used in the genetic diagnosis of VEOIBD ^63, 64^.

### Genotype-phenotype correlations

The best-described genotype-phenotype associations in IBD are *NOD2* variants and ileal CD ^65– 67^. Recent evidence has also pointed to UC-associated polymorphisms and phenotype associations. MHC-associated variants are of notable interest. As mentioned earlier, polymorphisms in the MHC region have been associated with extensive colonic involvement and colectomy ^33, 68^. Recently, the international IBD Genetics Consortium (IIBDGC) reported the results of the largest genotype-phenotype study to date, incorporating data from more than 34,000 IBD patients. Results pointed to three loci associated with disease subphenotype: *NOD2*, MHC, and 3p21. MHC loci were again associated with extensive colonic involvement ^69^.

### Prognosis

The ability to predict disease course at diagnosis as well as early identification of patients who will develop severe and medically refractory UC may result in changes in treatment algorithms.

Haritunians *et al.* developed a risk score utilizing a GWAS approach in a well-characterized cohort of patients with medically refractory UC. A modeled risk score using 46 SNPs explained 48% of the variance for colectomy risk in the population studied, with a sensitivity of 79% and a specificity of 86%. Further, this study confirmed associations of severe UC with SNPs within the MHC region, as well as SNPs in *TNFSF15*, *IL-10*, *IL-12B*, 12q15, *ZPF90, KIF1A*, and *GSDML/ORMDL3* ^70^. A study using the same strategy incorporated data from 703 UC patients from 40 sites within the IIBDGC. This study replicated an association between rs2403456 (11p15.3) with medically refractory, severe UC ^71^.

In order to identify genetic predictors of response to biologic therapy, Arijs *et al.* studied colonic mucosal gene expression signatures from two cohorts of anti-tumor necrosis factor (anti-TNF)-naïve UC patients receiving biologic induction with infliximab. Microarray-based colonic biopsy profiling was used to develop a prediction response probe set. Five genes − osteoprotegerin, stanniocalcin-1, prostaglandin-endoperoxide synthase 2, IL-13 receptor alpha-2, and IL-11 − were found to be differentially expressed in responders versus non-responders. The genes identified are involved in adaptive immune response signaling and TNF pathways ^72^. Of note, osteoprotegerin has been suggested as a stool marker for diagnosis and assessment of treatment response in pediatric UC ^73, 74^.

### Therapeutics

The intent of our discussion previously on genetics and UC is seeded in the hope that personalization of therapies based on genetics will change disease outcomes and potentially lead us to a cure of UC in the future. Though corticosteroids and mesalamine remain the mainstays of current therapy, the following sections will outline other options within our armamentarium with a special focus on the resurgence of drugs like methotrexate, the debate around antibiotics, and a brief look at potential newcomers.

Methotrexate, an immunosuppressant developed in 1948, has been widely used initially in leukemia and now in oncologic, rheumatologic, and autoimmune diseases. Methotrexate inhibits lymphocyte proliferation as a folate antagonist ^75^. Methotrexate has recently garnered attention as an alternative oral or subcutaneous agent in the treatment of UC, particularly in patients who have failed or become intolerant to 6-mercaptopurine (6-MP) or infliximab therapy. Two Cochrane reviews from 2014 and 2015, assessing induction and remission success, respectively, showed no significant difference between methotrexate and placebo in patients with UC ^76, 77^. Currently there are two large prospective trials evaluating the potential of methotrexate in UC. The Comparison of Methotrexate versus Placebo in Corticosteroid-dependent UC (METEOR) trial has completed enrollment and is closed. The Methotrexate Response In the Treatment of UC (MERIT-UC) trial is currently recruiting as multicenter prospective trials comparing methotrexate to placebo in patients who are steroid dependent or who failed 6-MP or infliximab therapy.

Antibiotics present an interesting clinical discussion in the setting of UC. Dysbiosis is present in patients with IBD, even at diagnosis. Previous work has shown that in patients with acute severe colitis, those with a more diverse microbiome at diagnosis respond better to steroid therapy versus those with a less diverse microbiome needing salvage medical therapy ^78^. A recent paper by Turner *et al.* described the use of an oral broad-spectrum antibiotic cocktail (metronidazole, amoxicillin, doxycycline, and vancomycin [MADoV]) in pediatric UC patients who had failed other traditional therapies (steroids, oral immunosuppressants including azathioprine and tacrolimus, and anti-TNF agents, both infliximab and adalimumab). A total of 7 out of 15 patients achieved complete short-term remission with the antibiotic cocktail. A larger pediatric randomized controlled trial to assess this intervention is underway ^79^. Additionally, in the discussion of dysbiosis, fecal microbial transplantation (FMT) has been proposed in the treatment of UC. There are several active recruiting studies ongoing; however, the only published data on the subject by Kunde *et al.* suggested a promising response in a pilot study of 10 children over 1 month ^80^.

The latest treatment for UC is a humanized monoclonal antibody named vedolizumab. An α4β7 integrin blocker, the medication targets inflammation by blocking the recruitment of lymphocytes specifically to the gut without interfering with trafficking to the central nervous system. This gut-specific feature makes it unique to the current group of therapeutics by isolating the affected area of involvement and subsequently decreasing the systemic effects ^81^.

Novel therapeutic targets continue to be developed and there are multiple drugs in different phases of development ( Figure 2).

**Figure 2.**
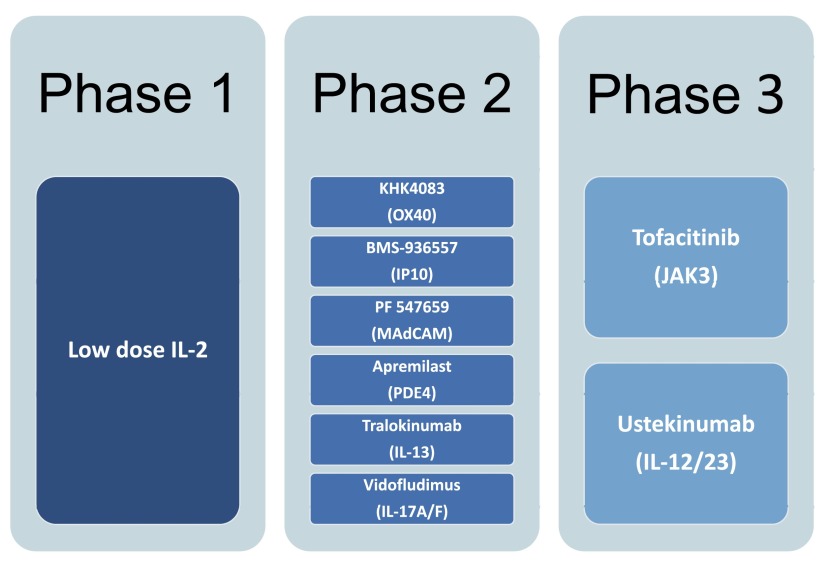
New therapeutic agents for ulcerative colitis. New therapeutic agents for ulcerative colitis with drug targets in parenthesis ^47, 83– 90^.

Treatment algorithms continue to evolve in UC and are increasingly utilizing predictive models to determine the best potential therapies for an individual patient. An integral part of the predictive model will be our continued advancement of the understanding of genetic data. Additionally, clinical variables have been recognized to impact the determination of initial therapy and as a predictive tool for choosing a more aggressive therapy in patients at risk for a poor outcome. Schechter *et al.* described the importance of the PUCAI score at baseline and moreover at the 3-month mark. The 3-month PUCAI was most predictive of sustained response and steroid-free remission at 1 year. Several other variables, including elevated C-reactive protein (CRP), hypoalbuminemia, and anemia have also been described in other studies, though not found to be significant in the Schechter model ^82^.

### Cure

Although a cure for polygenic complex diseases is still a hope, lessons from VEOIBD provide proof of principle that, indeed, certain well-characterized monogenic forms of IBD may be amenable to cure. Glocker *et al.* described a series of patients with infantile fistulizing enterocolitis resulting from mutations in *IL-10RA* and *IL-10RB*. Allogeneic stem cell transplantation in one of the patients resulted in complete resolution of gastrointestinal symptoms ^50^. Similarly, Worthey *et al.* described a patient with onset of fistulizing colonic disease at 15 months of age. Exome sequencing of this patient pointed to hemizygous missense mutation affecting *XIAP*; cord blood hematopoietic stem cell transplantation led to disease remission ^52^.

## Conclusions

The future of UC genetics looks bright, but there are a few hurdles to overcome. Current GWAS/Immunochip approaches are likely to have exhausted loci discovery in European ancestry populations; further multi-ethnic studies might lead to fruitful discoveries. Fine mapping and next-generation sequencing will provide additional tools for IBD-associated gene discoveries and may help explain some of the missing heritability in IBD, as well as provide a basis for functional studies that will in turn pave the way for targeted drug development.

Lastly, it is important to consider how genetics will influence daily clinical practice in the years to come and how clinicians will choose to adopt newer technologies. Certainly, the large amount of data associated with genetic information may be daunting to the general gastroenterology practitioner, and it is the job of researchers, IBD specialists, and working guideline groups to provide tools for daily clinical use.

As we look to the future, we can expect increasing integrated approaches using several biological disciplines to inform how genetic interactions shape and mold the pathogenesis and therapeutics of UC.
